# The signalling conformation of the insulin receptor ectodomain

**DOI:** 10.1038/s41467-018-06826-6

**Published:** 2018-10-24

**Authors:** Felix Weis, John G. Menting, Mai B. Margetts, Shu Jin Chan, Yibin Xu, Norbert Tennagels, Paulus Wohlfart, Thomas Langer, Christoph W. Müller, Matthias K. Dreyer, Michael C. Lawrence

**Affiliations:** 1 European Molecular Biology Laboratory (EMBL), Structural and Computational Biology Unit, Meyerhofstraße 1, 69117 Heidelberg, Germany; 2 The Walter and Eliza Hall Institute of Medical Research, 1G Royal Parade, Parkville, Victoria 3015 Australia; 3 Department of Medicine, University of Chicago, Chicago, IL 60637 USA; 4 Department of Medical Biology, University of Melbourne, Royal Parade, Parkville, Victoria 3050 Australia; 5 Sanofi-Aventis Deutschland GmbH, TA Diabetes and Integrated Drug Discovery, 65926 Frankfurt am Main, Germany; 6 Present Address: 6515 North Knox Avenue, Lincolnwood, Illinois 60712 USA

## Abstract

Understanding the structural biology of the insulin receptor and how it signals is of key importance in the development of insulin analogs to treat diabetes. We report here a cryo-electron microscopy structure of a single insulin bound to a physiologically relevant, high-affinity version of the receptor ectodomain, the latter generated through attachment of C-terminal leucine zipper elements to overcome the conformational flexibility associated with ectodomain truncation. The resolution of the cryo-electron microscopy maps is 3.2 Å in the insulin-binding region and 4.2 Å in the membrane-proximal region. The structure reveals how the membrane proximal domains of the receptor come together to effect signalling and how insulin’s negative cooperativity of binding likely arises. Our structure further provides insight into the high affinity of certain super-mitogenic insulins. Together, these findings provide a new platform for insulin analog investigation and design.

## Abstract (web summary)

The insulin receptor plays a key role in many physiological processes, yet how insulin effects receptor signaling at the structural level remains incomplete. Here the authors present a high-resolution cryo-EM structure of a high-affinity form of the insulin-bound insulin receptor ectodomain that sheds light on the mechanism of signal transduction.

## Introduction

**Fig. 1:**
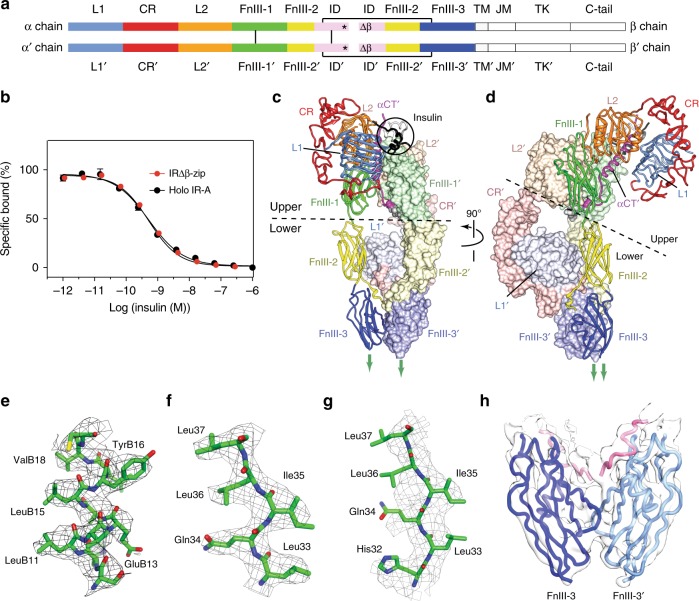
Insulin receptor structural biology. **a** Receptor domain layout^5^. L1 and L2: first and second leucine-rich repeat domains, CR: cysteine-rich domain, FnIII-1, -2 and -3: first-, second and third fibronectin Type-III domains, ID: insert domain, asterisk: α-chain C-terminal region (αCT), TM, JM: trans- and juxta-membrane domains, TK: tyrosine kinase domain, C-tail: β chain C-terminal segment. Black lines: inter-chain disulfide bonds; Δβː location of the glycosylated segment mutated/deleted in IRΔβ^5^. Domains within the second αβ polypeptide are distinguished by a prime (′) symbol. **b** Competition-binding curve for insulin binding to IRΔβ-zip immuno-captured from conditioned cell-culture medium (IC_50_ = 0.55 ± 0.02 nM; *n* = 4 technical replicates) compared to that for hIR-A immuno-captured from solubilized cell membranes (IC_50_ = 0.46 ± 0.06 nM; *n* = 4 technical replicates). Error bars (s.e.m.) are smaller than marker size when absent. The logIC_50_ values are identical at the 95% degree of confidence based on an *F* test (*P* = 0.171; no. of degrees of freedom = 80). **c**, **d** Orthogonal views of cryoEM structure of IRΔβ-zipInsFv. Circle: location of insulin. Fv modules are not shown but are attached to the respective CR domains as in prior X-ray crystal structures^5,38^ (see Supplementary Figure 4). **e**–**h** Sharpened density derived from the focused maps, covering (respectively) segments of the insulin B chain, domain L1 (insulin-bound), domain L1′ (insulin-free), and the dimeric FnIII-3:FnIII-3′ assembly

The human insulin receptor is a homodimeric, disulphide-linked (αβ)_2_ receptor tyrosine kinase. Despite the significance of the receptor’s signaling in a number of major disease states, a complete, atomic-level understanding of the way in which insulin binds to the receptor and effects signal transduction has proved elusive^1^. An obstacle is that the isolated, soluble receptor ectodomain (sIR), the entity most amenable to structural biology investigation, lacks the high affinity and the negative cooperativity of insulin binding that is characteristic of the holo-receptor (hIR)^2,3^. In particular, sIR binds two insulin molecules with equal nanomolar affinity with no negative cooperativity, whereas hIR binds one insulin molecule with picomolar affinity^2^ and displays negative cooperativity between its binding sites^4^. A three-dimensional (3D) crystal structure of insulin-free (i.e., apo) sIR has been determined to 3.3 Å resolution^5,6^ as well as single-particle cryoEM 3D structures of sIR in complex, respectively, with two insulin molecules (4.3 Å resolution; denoted here as “sIR + 2”) and with one insulin molecule (7.4 Å resolution; denoted here as “sIR + 1”)^7^. However, within both the latter cryoEM structures, the use of sIR precluded determination of how the high affinity and negative cooperativity of insulin binding are afforded to hIR. Furthermore, domains FnIII-2′, FnIII-3, and FnIII-3′ are severely disordered (and hence unmodelled) within sIR + 1 and sIR + 2, precluding interpretation of the mechanism of transmembrane signal transduction^8,9^ (receptor domain nomenclature is defined in Fig. 1a). By contrast with sIR, the only extant views of the apo- and insulin-bound hIR are at low resolution, being obtained by negative-stain transmission electron microscopy and two-dimensional (2D) class averaging of nanodisc-embedded hIR, without 3D reconstruction^9^.

To overcome the above difficulties and to obtain a high-resolution 3D image of the receptor in its signaling-active conformation, we devised a construct termed “IRΔβ-zip” that consists of the receptor ectodomain followed by a 33-residue GCN4 leucine zipper segment at the C terminus of the β chain. The ectodomain component of IRΔβ-zip is based on the construct IRΔβ that was employed in the crystal structure determination of sIR^5,6^ and lacks a short, highly glycosylated segment near the N terminus of the native β chain. Critically, attachment of the GCN4 zipper segment restores high-affinity insulin binding to IRΔβ (as has been shown for its attachment to sIR^10^).

Subsequent single-particle cryoEM analysis of insulin-bound IRΔβ-zip reveals a structure that has insulin bound in a fashion similar to that within sIR + 1, but within which all six FnIII domains are resolved. The overall resolution of the structure (3.2 Å in the insulin-binding region and 4.2 Å in the membrane-proximal region) is significantly higher than both that of sIR + 2 and sIR + 1. Strikingly, the C-terminal FnIII-3 domains are seen to be brought into association—an event that in the context of hIR will have the capacity to effect trans-phosphorylation of the intracellular tyrosine kinase domains^8,11^.

## Results

### Production and characterization of IRΔβ-zip

IRΔβ-zip (Supplementary Table 1) was purified by insulin elution from an insulin-affinity column, then complexed with the variable domain module (Fv) of the anti-IR antibody 83-7^12^, and finally subjected to mild endoglycosidase H treatment—the latter two steps initially intended to aid crystallographic study of the complex^13^. Insulin remained bound to IRΔβ-zip throughout these steps (see Methods and Supplementary Fig. 1), with the final complex being termed “IRΔβ-zipInsFv”. The affinity of IRΔβ-zip for insulin was determined by pull-down from conditioned cell-culture medium and shown to be sub-nanomolar, i.e., comparable to that of the holo-receptor for insulin (IRΔβ-zip: IC_50_ = 0.55 ± 0.02 nM, hIR-A: IC_50_ = 0.46 ± 0.06 nM; Fig. 1b).

### Single-particle cryoEM analysis of IRΔβ-zip

IRΔβ-zipInsFv was analysed by single-particle cryo-EM and the resulting density map was used to build an atomic model of the entire complex (Supplementary Figs 2 and 3 and Supplementary Table 2). The resolution of the initial map was sufficient to resolve the receptor domains (Fig. 1c, d). Only a single insulin was seen attached to the complex, consistent with IRΔβ-zip mimicking the negative cooperativity of hIR, i.e. binding of one insulin molecule to IRΔβ-zip markedly decreases its affinity for a second insulin. Improved maps were then obtained by focused refinement of “upper” and “lower” volumes of the complex, the upper volume encompassing domains L1, CR, L2, FnIII-1, L2′, FnIII-1′, αCT′, and insulin and the lower volume domains FnIII-2, FnIII-3, L1′, CR′, FnIII-2′, and FnIII-3′, with the polypeptide without the ′ symbol being that which contributes domain L1 to the insulin-binding site. Resolution of the resultant upper and lower maps was assessed as 3.2 Å and 4.2 Å, respectively, using the gold-standard Fourier shell correlation coefficient (FSC; Supplementary Fig. 3 and Supplementary Table 2). Representative volumes of the maps are given in Fig. 1e–h. The angular particle distributions are very similar for both maps (Supplementary Fig. 3), exhibiting some preferential orientations but, most importantly, exhibiting no “missing view” areas on the Euler sphere. In our judgment, the resolution discrepancy between the “upper” map and the “lower” map likely results from flexibility of the FnIII-2 and FnIII-3 domains within the “lower” part of the structure. Density corresponding to the Fv modules was apparent adjoining the known epitope on domains CR (Supplementary Fig. 4), but their associated volumes were excluded from the focused refinement as they showed signs of disorder. No density was apparent at any stage that could be attributed to the leucine zipper.

### Conformational change with respect to apo-receptor

**Fig. 2:**
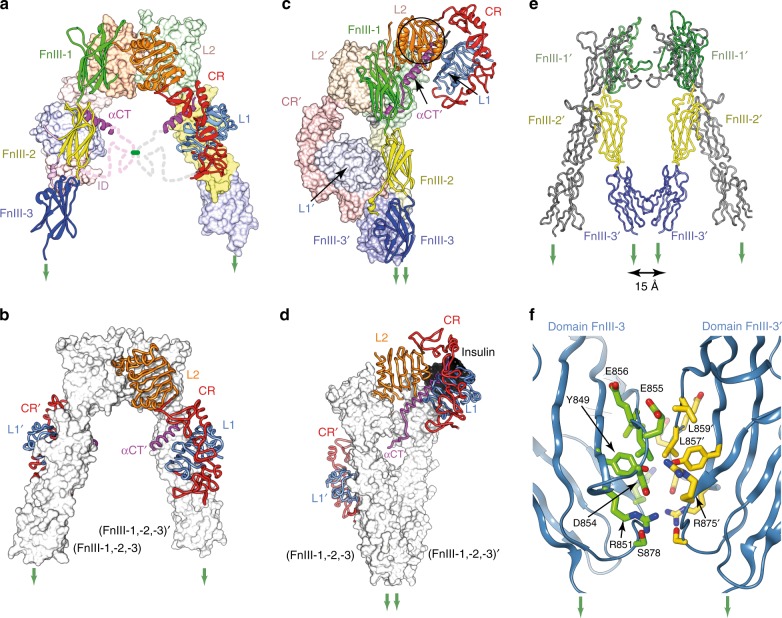
Comparison of the cryo-EM structure of IRΔβ-zipInsFv with the crystal structure of apo IRΔβ. **a** Domain arrangement in apo IRΔβ (left) with domains colored as in Fig. 1a. **b** Apo IRΔβ but with only domains L1, CR, L2, L1′, CR′, and αCT′ shown in color (for comparison with panel (**d**)). **c** Domain arrangement in IRΔβ-zipInsFv with domains colored as in Fig. 1a. **d** IRΔβ-zipInsFv but with only domains L1, CR, L2, L1′, CR′, and αCT′ shown in color in order to highlight the rearrangement of the insulin-bound (L1-CR-L2) + αCT′ module with respect to apo IRΔβ (panel (**b**)). **e** Comparison of the relative disposition of domains FnIII and FnIII′ in apo IRΔβ (grey) and in IRΔβ-zipInsFv (colored). **f** Pseudo-two-fold-symmetric interaction of domains FnIII-3 and FnIII-3′ within IRΔβ-zipInsFv. For clarity, only one member of each pseudo-symmetry-related residue pair is labeled. Green arrows in all panels indicate the direction of membrane entry. Panel (**a**) is adapted from Fig. 1a of Xu et al.^22^

Significant conformational changes are seen in IRΔβ-zipInsFv with respect to apo sIR. Strikingly, the fibronectin domain modules, i.e., (FnIII-1,-2,-3) and (FnIII-1′,-2′,-3′) are no longer in a Λ-shaped arrangement;^5,6^ instead, they are folded inwards in a pincer-like fashion that brings domains FnIII-3 and FnIII-3′ into contact (Fig. 1c, d and Fig. 2a–f). The association is effectively symmetric and occurs between the canonical C and C′ strands and EF loop of FnIII-3^14^ and their counterparts in FnIII-3′, involving residues Tyr849, Arg851, Asp854, Glu855, Glu856, Leu857, Leu859, Arg875, and Ser878 of each domain (Fig. 2f). Critically, we note that the distance between the respective most-C-terminally resolved residues of domains FnIII-3 and FnIII-3′ (i.e., Asp907 and Asp907′) is ~15 Å (Fig. 2e), well short of the maximum (~70 Å) that would be permitted for the total of eighteen residues that connect these termini to the N termini of the leucine zipper segments of IRΔβ-zip. The FnIII-3:FnIII-3′ association is thus not a hard constraint of zipper attachment. Indeed, a transition upon insulin binding in hIR from the open Λ-shaped structure of apo sIR to one in which the FnIII-domain modules have been brought together has been recently imaged at low (negative-stain electron-microscopy) resolution^9^. The bringing together of the FnIII-3 domains by insulin binding seen here is thus proposed to be the key event that concomitantly brings together the intracellular tyrosine kinase domains^8,11^ to permit trans-phosphorylation and initiation of the signaling cascade.

**Fig. 3:**
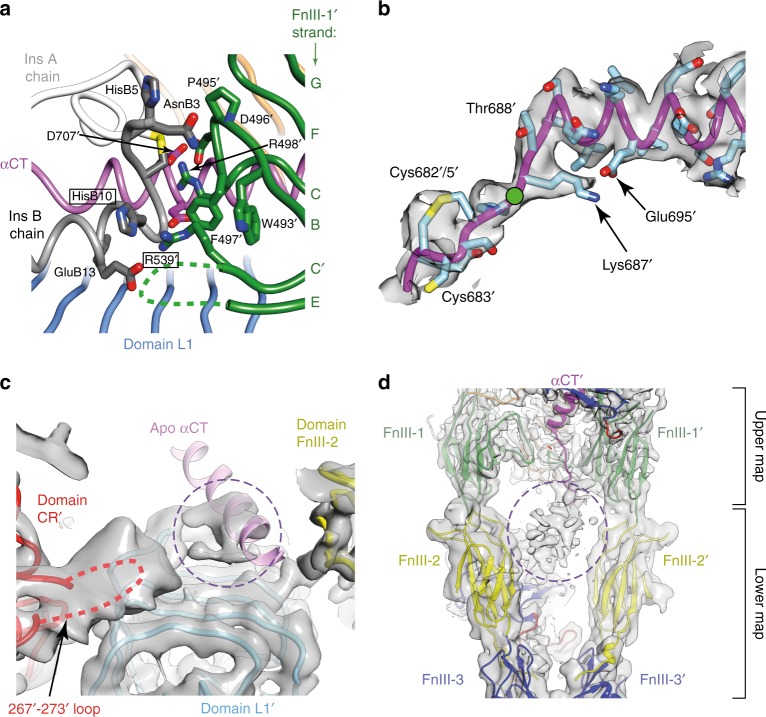
Detail of the cryo-EM structure of IRΔβ-zipInsFv. **a** Interaction of insulin with FnIII-1′, showing in particular the interaction of IR Arg539′ with insulin HisB10 (boxed residue labels). The dashed green line represents the poorly ordered segment between residues Ser541′ and Ser545′. **b** N-terminal region of the αCT′ helix showing termination of the helix at Asp689′ and the formation of a possible salt bridge between Lys687′ and Glu695′, as well as a possible conformation for the Cys 682′:Cys683′:Cys685′ triplet. Density shown is from the upper map. The green circle indicates the effective location of the four-glycine insert in the 686G4 mutant receptor and receptor ectodomain (see main text). **c** Density extracted from the lower map in the vicinity of the insulin-free domain L1, showing the density feature on the central β sheet of the domain (circled) and the overhanging CR′ domain loop containing residues Lys267′ to Gly273′ (dashed red line). The relative location of the αCT helix in the crystal structure of apo IRΔβ is shown for reference and is not included in the atomic model of IRΔβ-zipInsFv. **d** Density features (circled) associated with the unmodelled and partially disordered segments of ID and ID′. Density shown is extracted from both the upper and lower maps (as indicated on the right of the panel)

**Fig. 4:**
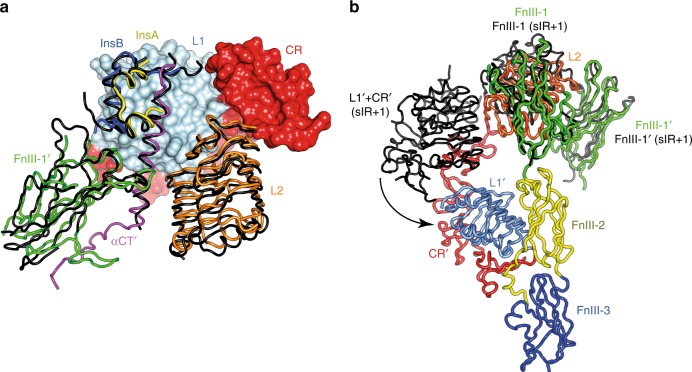
Comparison of the cryo-EM structure of IRΔβ-zipInsFv with the insulin-complexed cryo-EM structures sIR + 2 and sIR + 1^7^. **a** Schematic illustrating the similar environments of insulin, based on an overlay of the common L1-CR modules of IRΔβ-zipInsFv and sIR + 2 (shown as surface for IRΔβ-zipInsFv and omitted for sIR + 2). Remaining domains are shown as colored coil for IRΔβ-zipInsFv and black coil for sIR + 2. **b** Schematic illustrating the altered disposition of the respective unliganded L1-CR modules in IRΔβ-zipInsFv and in sIR + 1. Overlay is based on common FnIII-1/FnIII-1′ modules. Domains are shown as colored coil for IRΔβ-zipInsFv and black coil for sIR + 1

The insulin-complexed L1-CR-L2 module itself is folded out of the [L2-(FnIII-1):L2′-(FnIII-1′)] module of the receptor (Fig. 1c, d and Fig. 2c, d), re-positioning the insulin-bound L1 + αCT′ tandem element^15^ in such away that insulin makes contact with residues that lie within the canonical (and membrane-distal) BC and C′E loops^14^ of domain FnIII-1′ **(**Fig. 3a). While such re-positioning is similar to that seen in the cryoEM structures sIR + 2 and sIR + 1^7^ (Fig. 4a), the higher resolution of our structure clarifies detail of the hormone’s interaction with FnIII-1′ (Fig. 3a). In particular, an interaction is discerned between Arg539′ and insulin residue HisB10, with the side chain of Arg539′ stabilized by interactions with that of Trp493′ and Phe497′. Mutation of insulin HisB10 to acidic residues is known to result in insulins with slower off-rates and mitogenic signaling properties^16,17^. A reduced off-rate would be consistent with the formation of a salt bridge between the mutant Asp/GluB10 and Arg539′. The insulin-bound αCT′ helix is extended (with respect to its structure in apo sIR) in an N-terminal direction back to residue Asp689′, with the polypeptide being traceable back to the triple-Cys motif at residues 682′/683′/685′ (Fig. 3b). In the cryoEM structure sIR + 2^7^, the helix is also N-terminally extended, but only reportedly back to residue Ile692, suggesting that here it is in a yet more stable conformation.

The (insulin-free) domain L1′ is observed in our structure to retain an apo-like association with domain FnIII-2 (Fig. 2c, d). By contrast, domain L1′ in the 7.4 Å structure of sIR + 1 is located in the vicinity of, though not interacting with, domain FnIII-1^7^ (Fig. 4b). Furthermore, domain L1′ has here, on the surface of its central β-sheet, a large density feature (Fig. 3c) which we interpret as likely arising from the αCT segment seen in that location in the crystal structure of apo IRΔβ. The density is, however, poorly resolved and its longer axis is offset somewhat with respect to that of the αCT helix in apo IRΔβ. Also apparent in the vicinity is density associated with the loop formed by residues 265-275 within domain CR′. This loop, unmodelled here due to its partial disorder, appears to contact the αCT segment (Fig. 3c). Taken together, these latter features suggest a partial destabilization of the unoccupied primary insulin-binding site formed by the L1′ + αCT tandem element.

Several density features are apparent in the vicinity of the pseudo two-fold axis that relates the pair of FnIII-2:FnIII-3 modules (Fig. 3d). These features likely arise inter alia from the components of α- and α′-chain insert domains that connect the αCT and αCT′ segments to the respective upstream residues Cys647 and Cys647′. Residues Cys647 and Cys647′ and their respective disulfide-bond connections to β-chain residues Cys860 and Cys860′ are relatively well resolved in the density maps. Within the axial density features also lie the inter-α-chain disulfide bond(s)^18^ at residues Cys682/Cys683/Cys685 and Cys682′/Cys683′/Cys685′; however, these latter disulfide bond(s) and their interconnection cannot be unequivocally resolved.

The Fv 83-7 epitope (on domain CR) is distal to the insulin-binding sites both in the current and all prior structures of this receptor ectodomain^5,7^ and the Fv 83-7 itself is not here in proximity to any domain-domain interface within the insulin-bound IRΔβ-zip. The location of this moiety is such that there is no *prima facie* way in which it likely could have restricted or directed the conformational change associated with insulin binding, compatible with experiments that show that mAb 83-7 itself has no significant effect on insulin binding to hIR^19^. We conclude the structure presented here is most likely effectively identical to that which would be obtained without Fv attachment.

## Discussion

**Fig. 5:**
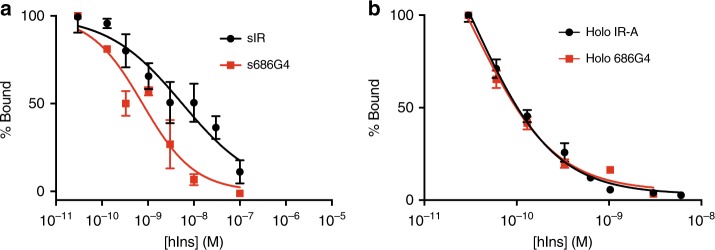
Insulin competition-binding assays for 686G4 IR mutants. **a** Insulin competition-binding assay of sIR (IC^50^ = 5.6 ± 1.7 nM; *n* = 4 technical replicates per concentration point) and s686G4 (IC_50_ = 0.76 ± 0.16 nM; *n* = 4 technical replicates per concentration point). The IC_50_ values differ at the 95% level of confidence based on an *F* test (*P* < 0.0001; no. of degrees of freedom = 54; two individual measurements of sIR affinity were judged as systematically in error and omitted from the analysis). **b** Insulin competition-binding assay of hIR-A (IC_50 _= 52 ± 10 pM; *n* = 4 technical replicates per data point) and holo 686G4 (IC_50_ = 30 ± 6 pM; *n* = 10 technical replicates per data point). The IC_50_ values are the same at the 95% level of confidence based on an *F* test (*P* = 0.084; no. of degrees of freedom = 86). In both (**a**) and (**b**), error bars reflect s.e.m. and are not shown when smaller than marker size

The structure presented provides insight not only into the signaling conformation of the receptor ectodomain but also into the nature of insulin’s second site of interaction on the receptor surface, seen here as involving domain FnIII-1′. It is tempting to map insulin’s interaction with domain FnIII-1′ (Fig. 3a) directly to that which affords hIR higher insulin affinity than sIR, as it has always been postulated that it is the presence of such an interaction that affords hIR’s higher insulin affinity^20^. However, the insulin interaction seen here with domain FnIII-1′ is also present in structures sIR + 1 and sIR + 2 (or to at least the degree discernible at the disparate spatial resolution of the three structures)^7^, but sIR itself does not display high-affinity (i.e., sub-nM) insulin binding^3,7^. The source of sIR’s lower insulin affinity is thus not obvious, but it may arise from the relatively large separation of the FnIII domains “legs” of apo sIR. Within apo sIR, the distance between the FnIII-2 and FnIII-2′ domains would regulate the distance between the αCT and αCT′ segments, as the latter are linked via disulfide bond(s) between the Cys682/Cys683/Cys685 triplets. We thus posit that there may be insufficient freedom in the disulfide-linked αCT segments of sIR to allow these segments to adopt conformations consistent with high-affinity insulin complexes at either or both the respective L1 + αCT′ and L1′ + αCT sites, yielding instead lower insulin affinity and an associated linear Scatchard plot^3^. Within apo IRΔβ-zip, zipper tethering results in a smaller separation of the FnIII-domain modules, allowing a single insulin bound to L1 + αCT′ sufficient conformational freedom to form a high-affinity complex. To test this hypothesis, we constructed a mutant sIR with four glycines inserted between Pro686 and Lys687 (location is indicated in Fig. 3b) and found that the mutant sIR (“s686G4”) bound insulin with significantly higher affinity (i.e., sub-nM) than sIR (see Methods and Fig. 5a). The affinity of hIR itself was not altered by the insertion of the four glycines (Fig. 5b). The increased affinity of s686G4 supports our hypothesis that adequate αCT conformational flexibility is necessary for high-affinity binding. Further (though indirect) support arises from the fact that the isolated ectodomain (sIGF-1R) of the holo type 1 insulin-like growth factor receptor (holo IGF-1R, a close homolog of the insulin receptor) binds insulin-like growth factors with sub-nM, holo IGF-1R-like affinity^21^, aligning with the observed smaller separation of the FnIII-domain modules within the isolated IGF-1R ectodomain^22^.

The above reasoning and observations may also be used to inform the source of the long-known negative cooperativity of insulin binding to hIR^4^ and (as observed here) to IRΔβ-zip. Negative cooperativity in the current context implies that when one insulin molecule binds to domains L1, CR, FnIII-1′, and αCT′ as observed here, a second insulin molecule is prevented from binding, at least not with high affinity, to domains L1′, CR′, FnIII-1, and αCT. Our above study of the s686G4 mutant indicates that a possible source of such restriction is the disulfide linkage between the Cys682′/Cys683′/Cys685′ and Cys682/Cys683/Cys685 triplets^18^. Similarly, we note that, unlike that in sIR + 1, it appears here that the unliganded binding site in IRΔβ-zipInsFv is maintained in near apo-like configuration, with αCT located upon the L1′ domain surface and domain L1′ in contact with FnIII-2. In the presence of excess insulin, insulin could bind at this second (apo-like) site but in so doing likely perturb the already insulin-bound site via the disulfide coupling of their respective αCT elements. Such binding would thus lead to accelerated dissociation of the already bound insulin, compatible with the classical observations demonstrating the negative cooperativity of insulin binding to hIR^4^.

**Fig. 6:**
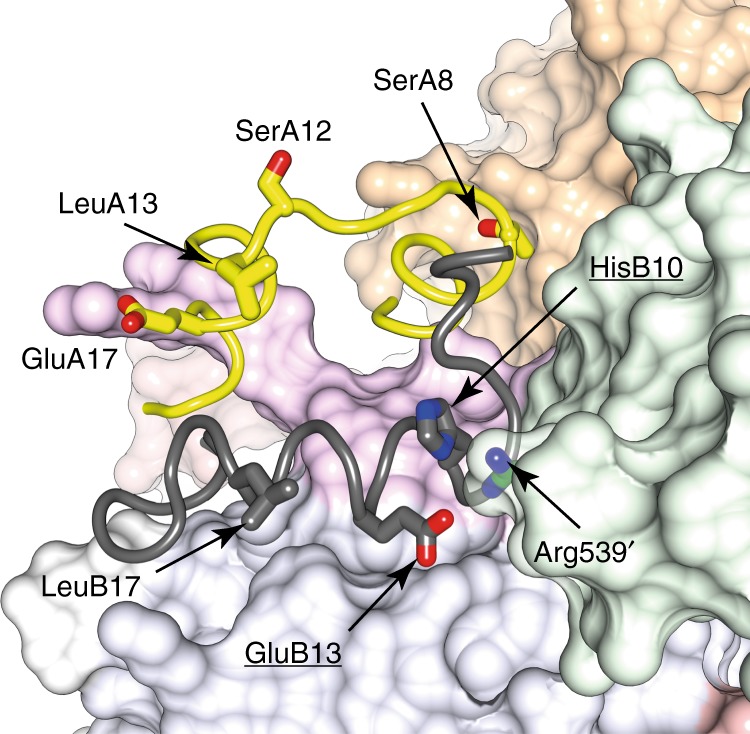
Disposition of insulin residues deemed to bind the second site on the receptor surface. Insulin is shown in ribbon representation (A chain: yellow; B chain: gray) and the surrounding receptor domains in molecular surface representation (L1: light blue; CR: light pink, L2: light orange; FnIII-1′: light green; αCT′: light magenta; N-linked glycan on domain L1: white). Insulin residues in stick representation are judged on the basis of alanine scanning mutagenesis^20,23^ to be involved in insulin’s interaction with its secondary binding site on the receptor surface. Of these, only His B10 and GluB13 (underlined labels) interact here with the receptor. Also shown is receptor residue Arg539′ that likely interacts with the carboxylate side chain present at position B10 in Asp/GluB10 mutant insulins (see Results for further discussion)

The second receptor-binding surface of insulin has been associated with the set of insulin residues SerA8, SerA12, LeuA13, GluA17, HisB10, GluB13, and LeuB17 that overlaps the hexamerization surface of insulin^20,23^. Of these residues, only HisB10 (and possibly GluB13) are observed here—and in structure sIR + 2—to interact with FnIII-1′, with insulin residues SerA12, LeuA13, GluA17, and LeuB17 being distal to that interaction and solvent exposed (Fig. 6). The role (if any) of SerA12, LeuA13, GluA17, and LeuB17 in receptor engagement thus lies elsewhere. We speculate that these residues may be involved in the initial docking of insulin to the receptor, an event postulated to precede the energetic relaxation of insulin’s induced fit to its primary ligand binding site^22^. Support for such a role at least for LeuA13 may come from the fact that its reduced affinity arises primarily from a reduced on-rate^24^. Understanding the manner of insulin’s initial engagement with its receptor might thus be the next frontier in the study of this system.

## Methods

### Cloning and production of IRΔβ-zip

A gene encoding IRΔβ-zip (residues 1-916 of the insulin receptor IRΔβ construct^5^ followed at its C terminus by the 33-residue GCN4 zipper sequence RMKQLEDKVEELLSKNYHLENEVARLKKLVGER^25^ and inclusive here of the population variants Tyr144His, Ile421Thr, and Gln 465Lys^26,27^) was synthesized by ATUM (USA) and then cloned into the pEE14 mammalian expression vector (Lonza) for stable expression of the protein in CHO Lec8 cells. Briefly, cells were transfected with complexes of plasmid DNA and X-tremeGENE 9 transfection agent (Roche) and then selected with 25 μM methionine sulphoximine in Lonza DMEM (High Glucose) medium containing 1 × GS supplement (Merck; i.e., “single strength”) and 10% dialysed fetal bovine serum (FBS; Life Technologies). Cells were plated in 96-well plates using limiting dilution and colonies allowed to form over several weeks. Secretion of target protein from colonies was detected via western blots using mAb 83-7 (hybridomas expressing mAb 83-7^12^ were a gift of Professor Ken Siddle, Cambridge, UK). Dozens of colonies were amplified in twelve-well trays and later in tissue culture flasks and monitored for expression of IRΔβ-zip. Several of the best-expressing clones were then screened further by seeding cells at exactly the same densities in six-well trays and individually monitored for expression over time. The single best-expressing clone was then selected to enter roller bottle scale-up. For scale-up, a richer medium containing 15% dialysed FBS and 2 × GS supplement (i.e., “double strength”) was used. Cells were seeded in roller bottles and allowed to grow for 14 days. 2.5 mM valproic acid (Sigma) was added at this point and the cells were allowed to incubate for a further 7 days, at which stage the conditioned medium was decanted from the roller bottles and filtered for purification.

### Assay of insulin binding to hIR-A and IRΔβ-zip

As a source of hIR-A for binding measurements (hIR-A = full-length construct of the insulin receptor isoform A), a Flp-In-CHO cell line (ThermoFisher Scientific; catalog no. R758-07) was transfected with a pcDNA 5/FRT/TO_Dest Plasmid (ThermoFisher Scientific) harboring the IR-A nucleotide sequence according to the manufacturer’s instructions. IR-A expressing cells were selected and maintained in medium containing hygromycin-B (300 µg mL^−1^). Cells were regularly demonstrated to be free of mycoplasma contamination using a kit (Lonza, MycoAlert) exploiting with high selectivity and sensitivity mycoplasmal enzymes in the cell-culture supernatants. Insulin competition-binding assay of insulin receptor constructs, (including isolation of membranes containing hIR-A and subsequent solubilization), were performed as follows^28^. A volume of 10 µg of a biotinylated anti-IR antibody 83-7 (ThermoFisher catalog no. MA5-13764) was added to streptavidin scintillation-proximity-assay (SPA) beads (5 mg) in 1000 mL binding buffer consisting of 100 mM HEPES–NaOH, 100 mM NaCl, 10 mM MgSO_4_, 0.025% (v/v) Tween-20 and adjusted to pH 7.8, and incubated for 30 min. After a single wash, a solution of solubilized hIR-A or IRΔβ-zip (provided as conditioned media from the initial cell culture, see above) was added and then incubated for further 60 min, followed by a single washing step. Subsequently, 100 μL re-suspended IR-antibody-SPA beads (containing 2–10 μg total insulin receptor) was mixed in a 96-well plate with 50 μL [^125^I]-labeled insulin tracer (100 pM, [^125^I]-(TyrA14)-insulin, Perkin Elmer) and 50 μL non-radioactive insulin (0.001–1000 nM). After incubation for 12 h at room temperature, radioactivity bound to the beads was measured in a microplate scintillation counter (Wallac Microbeta). Binding data were fitted using a nonlinear regression algorithm in GraphPad Prism7.0 (GraphPad Software Inc.).

### Affinity purification of insulin-bound IRΔβ-zip

For use in IRΔβ-zip purification, bis-BOC-insulin (BBI) affinity resin^3^ was prepared as follows. 1 gram batches of bovine insulin (bIns; Sigma catalog no. I5500) were individually suspended in 20 mL dimethyl sulfoxide (DMSO) and 1 mL triethylamine then added to raise the pH in order to dissolve the bIns. 74 mg of BOC (di-tert-butyl dicarbonate) dissolved in 1 mL DMSO was then added to the bIns, upon which the solution clarified. The mixture was incubated for 30 min on a rotator mixer at room temperature and the reaction then was stopped by addition of 50 uL ethanolamine. The modified bIns was collected by precipitation after addition of 400 mL ice-chilled acetone with the addition of several drops of concentrated HCl until a precipitate formed. The samples were incubated at 4 °C overnight and centrifuged to isolate the precipitate. This was repeated twice on the removed supernatant and the pelleted BOC-modified bIns was broken up and dried under a stream of nitrogen gas. A 50 cm diameter, 200 mL bed-volume cation-exchange column was then prepared using SP-Sepharose Fast Flow (GE Healthcare Life Sciences) and equilibrated with 1.5 M acetic acid, 7 M urea, pH adjusted to 3.0 with HCl (buffer A). BOC-modified bIns pellets were dissolved in 50 mL buffer A and after loading onto the column in several portions, variously-BOC-modified bIns fractions were eluted with gradients of buffer A to buffer B (1.5 M acetic acid, 7 M urea, 0.5 M NaCl, pH adjusted to 3.0 with HCl) as follows. The column was pre-loaded with 0.25 column volumes (CVs) of buffer A followed by injection of 9 mL BOC-modified bIns. The column was then washed with 1 CV of buffer A, followed by gradients of 0 to 10 % buffer B (0.25 CV), 10 to 25% B (4 CV), and 25 to 100% B (0.2 CV). The volume of the peak fractions putatively containing BBI (as opposed to mono- and tris-BOC bIns; putative peak identification kindly provided by Mr John Bentley (CSIRO; Parkville, Australia)^29^, see below for final mass spectroscopy verification) was then reduced by pooling and binding again to the column and eluting as follows. Approximately 3 L of fractions containing BBI were diluted 1:1 v/v with water, loaded in portions on the column and re-eluted with a single gradient of 200 mL 0 to 100% buffer B. Approximately 370 ml of combined BBI fractions were dialysed in two 40 cm long 54 mm diameter 3.5 kDa cut-off dialysis bags against 10 L pure water with more than twenty changes. BBI precipitated by dialysis was collected by centrifugation. Sedimented BBI pellets were dissolved in minimum volumes of 0.1% TFA/80% acetonitrile or 10 mM HCl and were freeze-dried. Total dry weight was 1.3 gm. N-terminal sequence analysis (Australian Proteome Analysis Facility; Sydney, Australia) revealed a major signal for the B-chain N terminus and a minor signal (13%) for the A-chain N terminus indicating that most of the A-chain N-terminal residues in the sample were BOC modified, as required^3^. Finally matrix-assisted laser desorption ionization time-of-flight mass spectrometry confirmed that most of the sample was of mass corresponding to BBI, with only small quantities of mono- and tris-BOC bIns present. Two 50 mL batches of Mini Leak Medium activated resin (divinyl-sulfone-activated agarose; Kem-En-Tec, Denmark) were then washed extensively on a sintered glass funnel with water and then with 100 mM sodium bicarbonate-sodium hydroxide, pH 8.5. The resin was transferred to a Schott bottle containing 60 mL of 100 mM sodium bicarbonate-sodium hydroxide, pH 8.5. One gram of BBI dissolved in DMSO was dropped slowly with frequent mixing into the resin slurry in an ice-water bath. The combined resin and protein mix was rocked in the Schott bottle at 32 °C for 6 days. Unreacted divinyl-sulfone-reactive groups were reacted with 200 mM ethanolamine-HCl (pH 9.0), after removal of BBI solution from settled resin. The resin was washed with 50% DMSO, then water and finally with tris-buffered saline plus azide (TBSA; 24.8 mM Tris-HCl (pH 8.0), 137 mM NaCl, 2.7 mM KCl, and 0.02% sodium azide) and finally stored in 20% ethanol for future use.

IRΔβ-zip was purified typically from a single 10 L batch of conditioned medium to which was added phenylmethylsulfonyl fluoride (PMSF; 1:1000 dilution of 100 mM PMSF/propan-2-ol; Merck), sodium azide (Sigma-Aldrich) to 0.02% and 5 mL of 3 M Tris-HCl, pH 8.5 per litre of medium. The medium was then filtered through a 0.2 μm bottle filter (Thermo Scientific) to remove insoluble material. Sample volume reduction and concentration was achieved by cycling it continuously at room temperature through a stack of two Pellicon 3, 0.11 m^2^, 10 kDa concentrator cartridges (Merck-Millipore) until the sample had been concentrated 5- to 20-fold (with FBS concentration rising from 15% to at least 100%). The concentrate was filtered through a 0.2 μm bottle filter and stored at 4 °C in the presence of an additional 0.05% sodium azide. For purification, the filtered concentrate was run through a 20-mL bed volume (BV), 50-cm diameter, Sepharose CL-4B (GE |Healthcare Life Sciences) guard column to remove non-specifically adsorbing material. IRΔβ-zip was captured on a 15- or 20 mL bed-volume BBI affinity column (BBI prepared as described above) by running the sample through under gravity. Unbound material was washed off the column with at least ten CVs of TBSA buffer. Weakly bound receptor and contaminants were eluted by running three CVs of 0.4 M NaCl, 0.2 M tri-sodium citrate-HCl (pH 5.0) through the column. Only a very small quantity of IRΔβ-zip was evident in this fraction on SDS-PAGE analysis. Receptor was eluted with two CVs of a solution of 50 μM human insulin (Sigma-Aldrich, catalog no. 91077C) in TBSA buffer, followed by two CVs of 5 μM human insulin in TBSA and then two CVs of TBSA. Sample pH was adjusted by the addition of one-third volume 3.0 M Tris-HCl, pH 8.5 and PMSF was added to 0.1 mM. The purification was repeated until the IRΔβ-zip yield dropped substantially, indicating depletion of the receptor from the medium. Insulin-eluted fractions were concentrated in either an Amicon 200 mL stirred cell with 10 kDa cut-off membrane or an Ultra-15 10 kDa centrifugal concentrator (Millipore) and then purified further by size-exclusion chromatography (SEC) using either a Superdex 200 10/300 GL or a Superdex S200 26/60 column (GE Healthcare Life Sciences) equilibrated with TBSA buffer. Relevant Coomassie-stained SDS-PAGE gel images and SEC profiles for the above steps are provided in Supplementary Fig. 1; insulin was seen to remain bound to IRΔβ-zip in the final product.

### Cloning and expression of Fv 83-7

Fv 83-7 was prepared using a *Brevibacillus* expression system using protocols identical to those described previously^13^. Briefly, codon- and expression-optimised DNA corresponding to murine monoclonal antibody 83-7 variable heavy (VH) chain residues 1-118^5,6,12^ followed by the sequence SLVPRGSSSEQKLISEEDLN (thrombin cleavage site + c-myc tag) was synthesized and then cloned into the vector pCDNA3.1 by DNA2.0 (USA). Similarly, DNA encoding the 83-7 variable light (VL) chain residues 1-112^5,6,12^ followed by the sequence SSDYKD (FLAG tag) was synthesized and then cloned into the vector pJ201 (DNA2.0, USA). Both genes were then individually transferred into the BamH1/Xba1 sites (in frame with a secretion signal) of the plasmid pNCM02 (Takara Bio, Japan) for independent transformation into *Brevibacillus choshinensis* cells (Takara Bio, Japan). Isolated colonies of the transformed *B. choshinensis* cells were then screened by western blot (antibodies 9E10 and M2, respectively) for overexpression of the expected domain. The highest-expressing colonies were then stored as glycerol stocks. For 1 L scale-up, each glycerol stock was used to inoculate 2 mL of 2SY broth containing 10 μg mL^−1^ neomycin sulfate (Sigma-Aldrich, USA) (2SYnm), followed by incubation overnight at 30 °C at 120 rpm. A volume of 0.2 mL of these respective cultures were then used to inoculate a further 20 mL of 2SYnm broth and, once sufficiently grown, 5 mL of this inoculum was used to inoculate a further 500 mL of 2SYnm broth in Tunair™ flasks (Sigma Aldrich, USA). Cultures were incubated for 72–96 h, with 1 mL samples taken at 24 h intervals to monitor production via SDS-PAGE and western blot. Optical density was monitored at 660 nm. Samples were centrifuged at 13,000 rpm for 5 min to pellet bacteria and to recover the supernatant containing the secreted product.

### Assembly and purification of Fv 83-7

A volume of 700 mL of conditioned medium containing the c-myc-tagged 83-7 VH domain was combined with 850 mL of conditioned medium containing FLAG-tagged 83-7 VL domain and incubated for 30 min at room temperature, followed by addition of 3 M Tris HCl (pH 8.5) at the ratio of 5 ml/L of combined media. This process was estimated to give a slight excess of VL monomers in the VL/VH mixture. The pH-adjusted combined media was then run through a 9E10 Mini-Leak Low affinity column (Kem-En-Tec, Denmark)^30^ and the desired Fv 83-7 eluted with c-myc peptide (decameric form) prepared in Tris-buffered saline plus azide (24.8 mM Tris-HCl pH 8.0, 137 mM NaCl, 2.7 mM KCl plus 0.02% NaN_3_; "TBSA"). Fractions were combined with one tablet of cOmplete protease inhibitor cocktail (Roche, Switzerland). Fractions from Superdex 200 10/300 SEC (GE Healthcare Life Sciences) examined by SDS-PAGE showed the presence of two bands of molecular weight 14 and 16 kDa, respectively, indicating a correctly-formed Fv eluting at 22 kDa. The c-myc tag was then removed from the 83-7 VH domain of the Fv 83-7 as follows. The Fv 83-7 was diluted to ~4 mg mL^−1^ in TBSA and then combined with 10 mM CaCl_2_ and 0.5 U human thrombin per mg Fv 83-7 (Roche, Switzerland). The sample was incubated at 37 °C for 4 h and the reaction stopped by the addition of 1 mM phenylmethylsulfonyl fluoride and by incubation on ice. The sample was then re-purified by means of a Superdex S75 column (GE Healthcare Life Sciences, USA). SEC and SDS-PAGE estimates of molecular weight agreed with the expected masses.

### Assembly of IRΔβ-zipInsFv

The insulin-complexed IRΔβ-zip was then mixed with 1.25 mol equivalent of Fv 83-7 in TBSA buffer. The sample was then incubated in Glyco buffer 3 with 10,000 U/mg of endoglycosidase H (NEB) for 26 h at 37 °C at ~5mg.mL^−1^ receptor. The degree of deglycosylation was assessed by SDS-PAGE analysis. The IRΔβ-zipInsFv sample was then centrifuged and run on a Superdex S200 10/300 SEC column (GE Healthcare Life Sciences) in TBSA buffer.

### Single-particle cryo-electron microscopy

3.6 μL aliquots of the IRΔβ-zipInsFv protein complex diluted to 340 nM in HEPES buffer (pH 7.5) were applied to holey carbon grids (C-flat 300 mesh R2/2; Protochips), glow discharged in a Pelco EasyGlow device. Grids were blotted and flash-frozen in liquid ethane using a Vitrobot mark IV (ThermoFisher) with a blotting time of 1 s at 10 °C and 100% humidity. Data acquisition was performed on a Titan Krios microscope (ThermoFisher) operated at 300 kV, through a Gatan Quantum 967 LS energy filter using a 20 eV slit width in zero‐loss mode. The data set was recorded on a Gatan K2‐Summit direct electron detector operated in super-resolution mode, at a calibrated magnification of ×130,000 (resulting in a super-resolution pixel size of 0.52 Å on the object scale) with a defocus range of 1–2.5 μm. The movies were recorded in 20 frames for the 16 sec exposures with a dose rate of 2.5 e^−^/physical pix/s, accumulating a total dose of 37 e^−^/Å^2^ at the sample level. Data collection was performed fully automatically on a single grid during a 48 h session using SerialEM^31^.

After visual inspection of the micrographs, 2287 images were selected for further processing. The movie frames were aligned and dose-compensated with MotionCor2^32^ using patch-based alignment (5 × 5) and Fourier space cropping (by a factor 2), resulting in a pixel size of 1.04 Å. Contrast transfer function parameters for the micrographs were estimated using Gctf^33^. Particles were picked automatically using RELION^34^, resulting in 747,074 raw particles. All 2D- and 3D classifications and refinements were performed using RELION^35–37^. After one round of 2D classification, 506,594 particles remained in the data set, in which only classes displaying “clean” complexes and high signal-to-noise ratio were selected. This particle data set was then submitted to 3D classification and sorted into eight classes, using as an initial model the symmetrized domains L1, CR, and L2 bound to Fv 83-7 from the crystal structure of the human insulin receptor ectodomain (PDB: 4ZXB)^6^ low-pass filtered to 60 Å. Two of the classes, representing 213,867 particles (42% of the data set) showed clear features of “closed” FnIII domains and were used for further 3D refinement. The resulting map allowed ready identification of the constituent domains of IRΔβ-zipInsFv based on the known crystal structure of apo IRΔβ in complex with Fab 83-7 and Fab 83-14^5,6,15^ and of the insulin-complexed μIR^38,39^. The “upper” part of the map (encompassing domains L1, CR, L2, FnIII-1, L2′, FnIII-1′, αCT′, and insulin) was further improved with a focused 3D refinement step using a soft mask encompassing this volume, excluding the “lower” part (encompassing domains FnIII-2, FnIII-3, L1′, CR′, FnIII-2′, and FnIII-3′) and the Fv modules. The reported overall resolution of 3.2 Å was calculated using the gold-standard Fourier shell correlation (FSC) 0.143 criterion^40^ and was corrected for the effects of a soft mask on the FSC curve using high-resolution noise substitution^41^. The final density map was corrected for the modulation transfer function of the detector and sharpened by applying a negative B factor that was estimated using automated procedures^42^.

The “lower” part of the map showed clear signs of heterogeneity, especially in the FnIII domains conformation. A subsequent, focused 3D classification step was performed after partial signal subtraction of the “upper” part^43^. Two of the classes, representing 98,481 particles, displayed a “closed” and homogeneous state of the FnIII domains and were used for further focused 3D refinement using a soft mask encompassing the “lower” part, excluding the “upper” part and Fv modules, leading to an overall resolution of 4.2 Å.

### Real-space refinement

Domains were identified by visual inspection of the cryo-EM map. Model-building was initiated by rigid-body docking of the individual crystal-structure-derived domains of IRΔβ (obtained from “Model S1” within ref ^6^., an extended version of Protein Data Bank entry 4ZXB) directly into the respective maps using CHIMERA v1.11.2^44^. Model-building into the upper map employed multiple rounds of manual editing in COOT v0.8.7^45^ and real-space refinement within PHENIX v1.13-2998-1692^46^ with secondary structure, rotamer and Ramachandran restraints applied throughout. A single (“grouped”) atomic displacement parameter (ADP) was assigned to each residue. Weighting was determined using the automated protocol within PHENIX. N-acetylglucosamine residues were included where visible^47^. A density feature of approximate diameter 12 Å was apparent within the upper map, its shape being approximately that of hollow sphere (Supplementary Figure 5); it was presumed to be a metabolite from the cell-culture medium and was left unmodelled as its identity could not be determined by mass spectrometry (data not shown). Model-building into the lower map employed no graphics-based manual model-building apart from residue deletion. Real-space refinement employed main- and side-chain torsional restraint against the extended dimeric form of the crystal structure of apo IRΔβ (i.e., “Model S1”, see above). N-acetylglucosamine residues were included where visible^47^. The stereochemical weight was set at 1.5 and the final refinement step included grouped ADP refinement. The statistics of the final refined models are given in Supplementary Table 2. Molecular graphics were generated using CHIMERA^44^.

### Production of hIR-A and sIR-A and their 686G4 mutants

Holo-receptor (hIR-A, residues 1-1343) and ectodomain (sIR-A, residues 1-917) sequences of the A isoform of the insulin receptor were cloned into pcDNA3.1 zeo( + ) (ThermoFisher Scientific). Both plasmids were mutagenized using the QuikChange kit (Agilent Technologies) to introduce codons corresponding to four glycines immediately after residue 686 using the following oligonucleotides: forward 5′-TGCTCCTGTCCAGGAGGTGGAGGTAAGACAGAC-3′ and reverse 5′- GTCTGTCTTACCTCCACCTCCTGGACAGGAGCA-3′. After mutagenesis, the complete cDNA sequences in all plasmids were verified by DNA sequencing prior to use. For the soluble receptor ectodomain constructs, HEK 293-H cells were transfected in six-well trays with wild type and mutated plasmids (686G4). Cell-culture media were collected 24–48 h post transfection and the receptor partially purified by affinity chromatography on wheat-germ agglutinin (WGA) agarose and eluted with 0.3 M *N***-**acetyl-D-glucosamine, 20 mM HEPES (pH 7.5), 150 mM NaCl, 0.1% Triton-X 100, 5% glycerol. For the wild type and 686G4 hIR-A constructs, transfected HEK 293-H cells were treated with 1 ml/well lysis buffer (0.1% Triton-X 100, 20 mM HEPES pH 7.5, 150 mM NaCl) plus cOmplete protease inhibitor cocktail (Roche). The extract was centrifuged briefly to remove debris and the receptor was partially purified by WGA affinity chromatography as above.

### Affinity analysis of hIR-A and sIR-A 686G4 mutants

Competition binding on insulin binding to 686G4 mutated insulin receptor was performed via a polyethylene glycol (PEG) precipitation assay. Receptor samples were incubated in 1.5 mL Eppendorf tubes for 16 h at 4 °C in a total volume of 100 μL with 30 pM [^125^I]-(TyrA14)-insulin (Perkin Elmer) together with unlabeled insulin at various concentrations in binding buffer (100 mM HEPES, pH 8.0, 100 mM NaCl, 10 mM MgCl_2_, 0.25% (w/v) BSA, 0.025% (w/v) Triton X-100). A volume of 0.1 mL Krebs-Ringer bicarbonate buffer containing 0.05% BSA, 500 μL 0.2% γ-globulin, and 500 μL of 25% (w/v) polyethylene glycol 8000 was added, samples were held on ice for 10 min, micro-centrifuged for 10 min, supernatants were removed by aspiration and the pellets were counted in a γ-counter. For competition experiments, the concentration of receptor was adjusted to yield less than 20% binding when no competing insulin was added. Binding data were fitted using a nonlinear regression algorithm in GraphPad Prism7.0 (GraphPad Software Inc.; see Supplementary Figure 5).

## Electronic supplementary material

Supplementary Information

## Data Availability

The coordinates of the refined models have been deposited in the Protein Data Bank (“upper” domains: PDB entry 6HN5; “lower” domains: PDB entry 6HN4) and the corresponding *B*-factor sharpened cryoEM maps from which they were derived in the Electron Microscopy Data Bank (“upper” domains: EMDB entry EMD-0247; “lower” domains: EMDB entry EMD-0246). Other data are available from the corresponding authors upon reasonable request.
