# Supplementary material for: The signalling conformation of the insulin receptor ectodomain
Source: Nat Commun. 2018 Oct 24;9:4420. doi: 10.1038/s41467-018-06826-6 (PMC6200814; doi:10.1038/s41467-018-06826-6)
Supplement: Supplementary file 1 — Supplementary Information [file 41467_2018_6826_MOESM1_ESM.pdf]

Supplementary Information

The signalling conformation of the insulin receptor ectodomain

Weis *et al.*

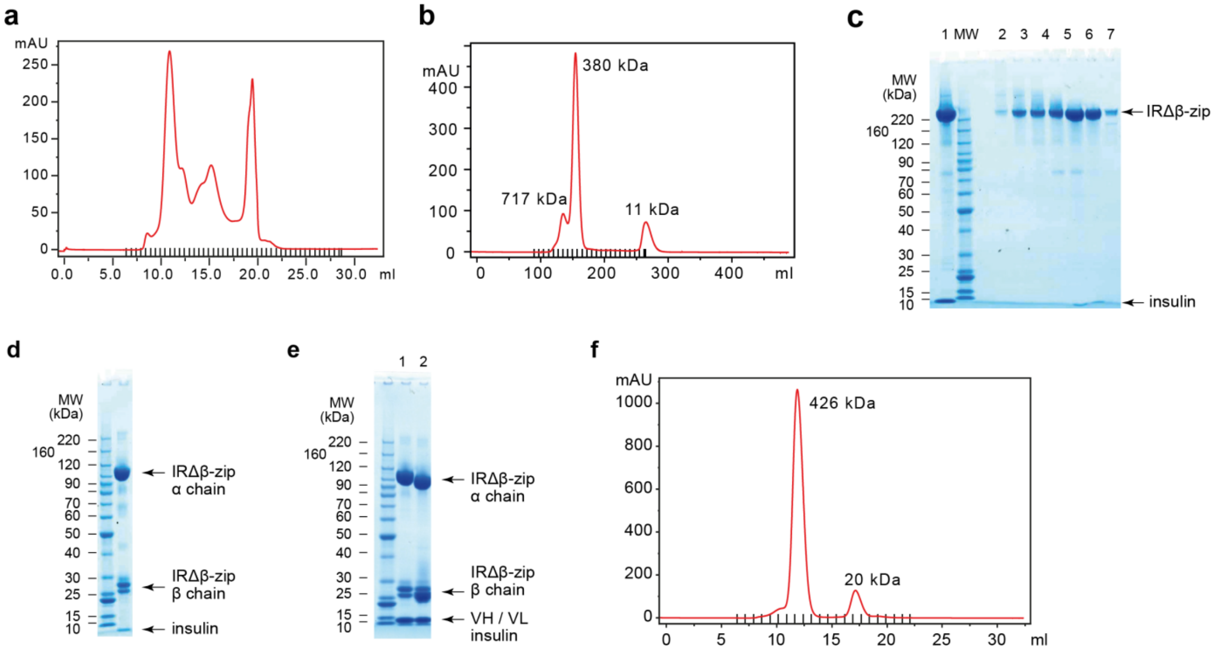

Supplementary Figure 1: Characterization of IRΔβ-zip and production and purification of IRΔβ-zipInsFv.

a, Size-exclusion chromatography (SEC) profile of protein eluted using a pH 5.0 buffer from the insulin-affinity column, showing extensive sample heterogeneity. **b,** SEC profile of protein subsequently eluted using insulin from the insulin-affinity column. Peaks from left to right correspond to $(\text{IR}\Delta\beta\text{-zipIns})_2$, IRΔβ-zipIns and insulin. **c,** Coomassie-stained SDS-PAGE gel with lanes 2-7 representing fractions spanning the 380 kDa peak in profile (b) and lane 1 the starting material for the SEC of the insulin-eluted material. All sample lanes are non-reduced. **d,** Coomassie-stained SDS-PAGE gel of the reduced peak fraction of profile (b). **e,** Coomassie-stained SDS-PAGE gel of reduced IRΔβ-zipInsFv before and after endoglycosidase-H treatment (lanes 1 and 2, respectively). In (d) and (e), the multiple bands for the β chain are assumed to arise from varying glycosylation patterns. **f,** SEC profile of IRΔβ-zipIns combined with an excess of Fv 83-7 and then treated with endoglycosidase H. The left-hand peak corresponds to the endoglycosidase-H-treated IRΔβ-zipInsFv and the right-hand peak to the excess of uncomplexed Fv 83-7.

a

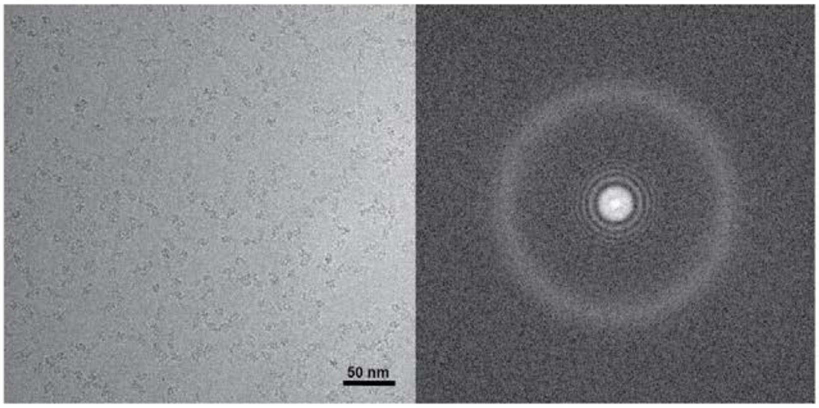

b

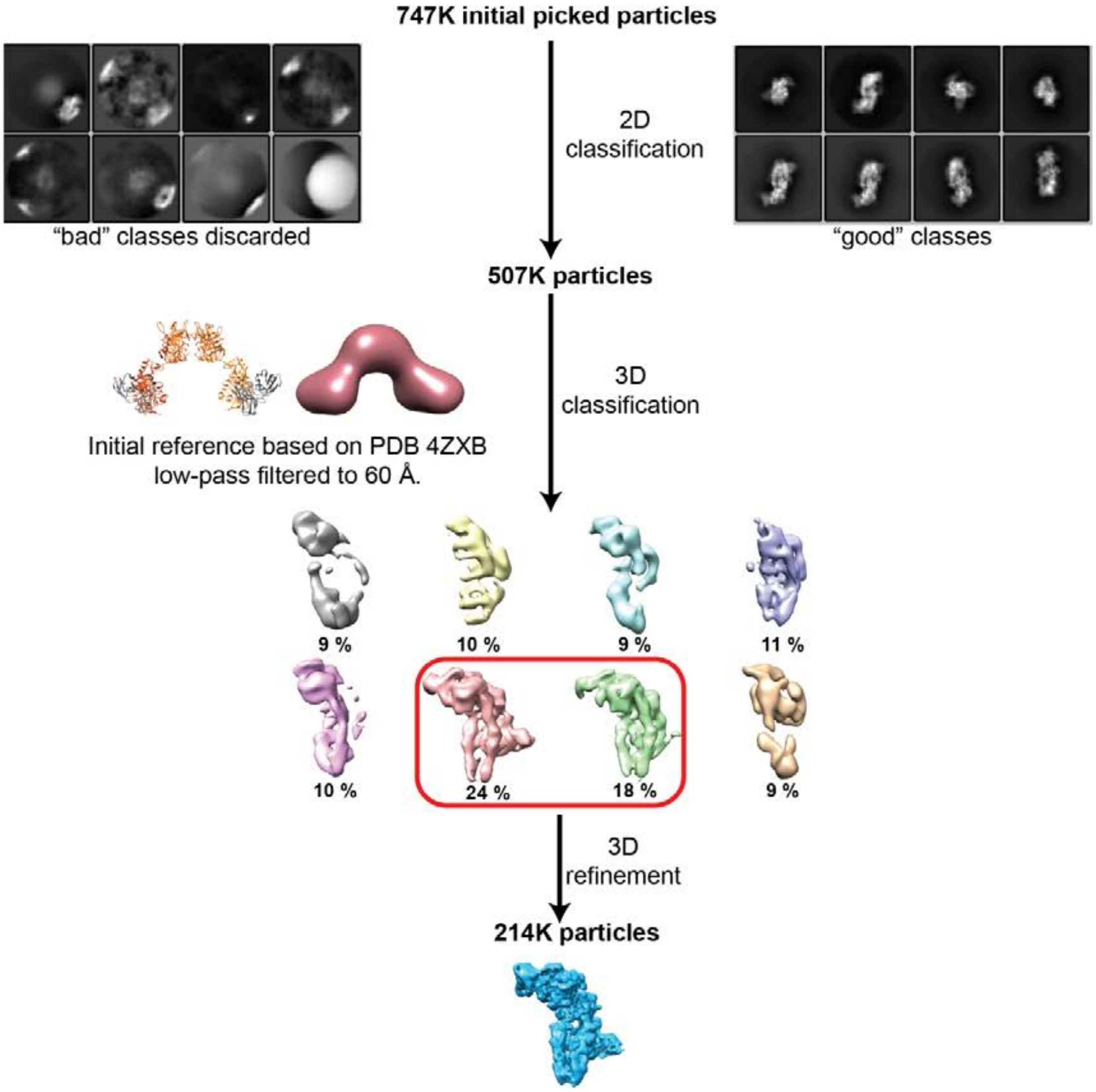

Supplementary Figure 2: Image processing workflow. **a**, Representative micrograph (1.04 Å / pixel) after whole frame alignment and corresponding power spectrum. Image generated using the *framewatcher* routine from IMOD[1]. **b**, Overview of the data processing workflow.

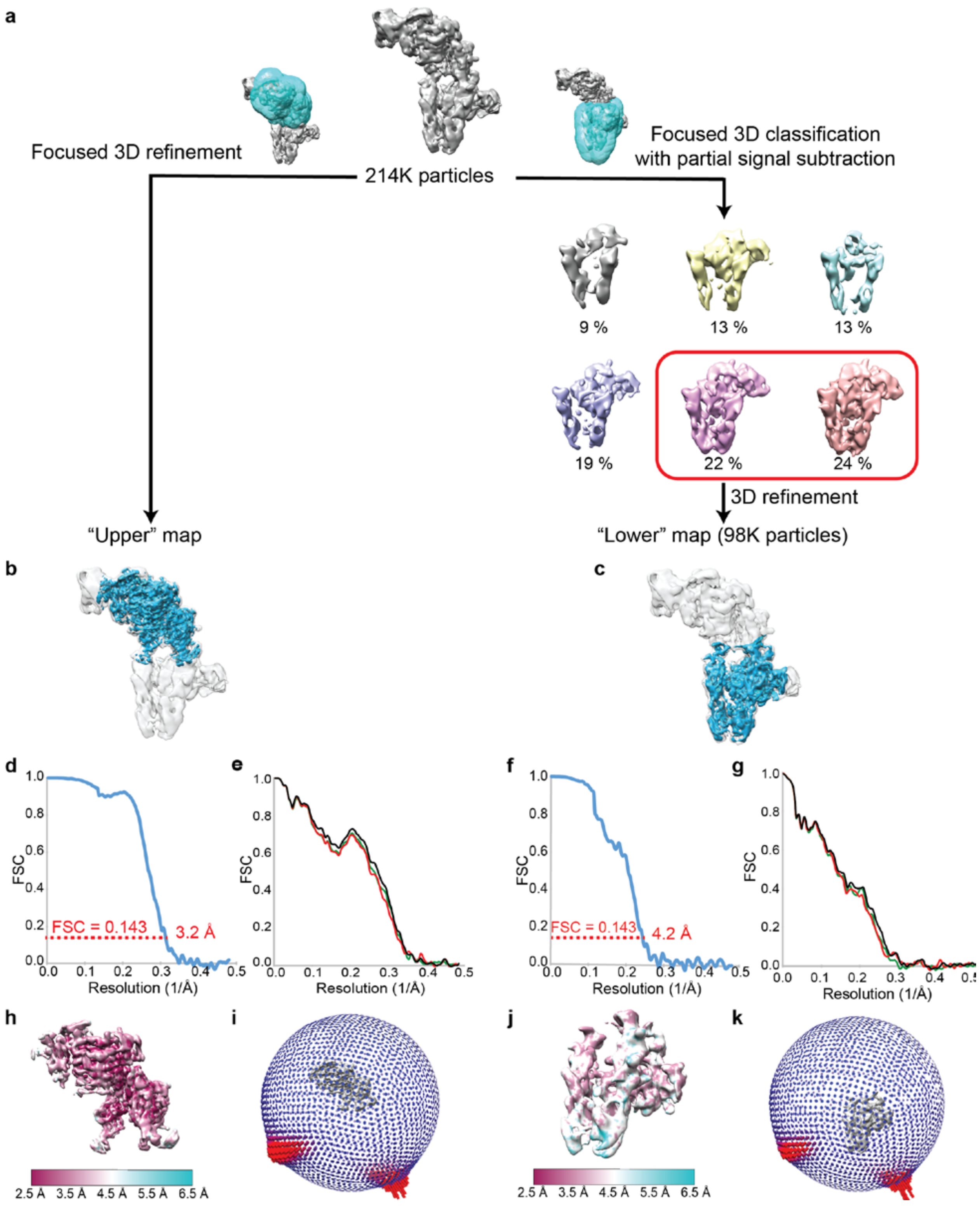

Supplementary Figure 3. Focused refinement and resolution estimation. **a**, Overview of the focused classification and refinement strategy used to generate the “upper” map, **b**, and the “lower” map, **c**. The soft mask used are shown in transparent cyan. **d** and **f**, Resolution estimation from the FSC curves. **e** and **g**, FSC curves between the refined structure and the map calculated from the full dataset (black), the half-map used in refinement (green), and the other half-map (red). **h** and **j**, Unsharpened final maps coloured according to local resolution calculated with the ResMap software package [2]. **i** and **k**, Euler angle distribution plots.

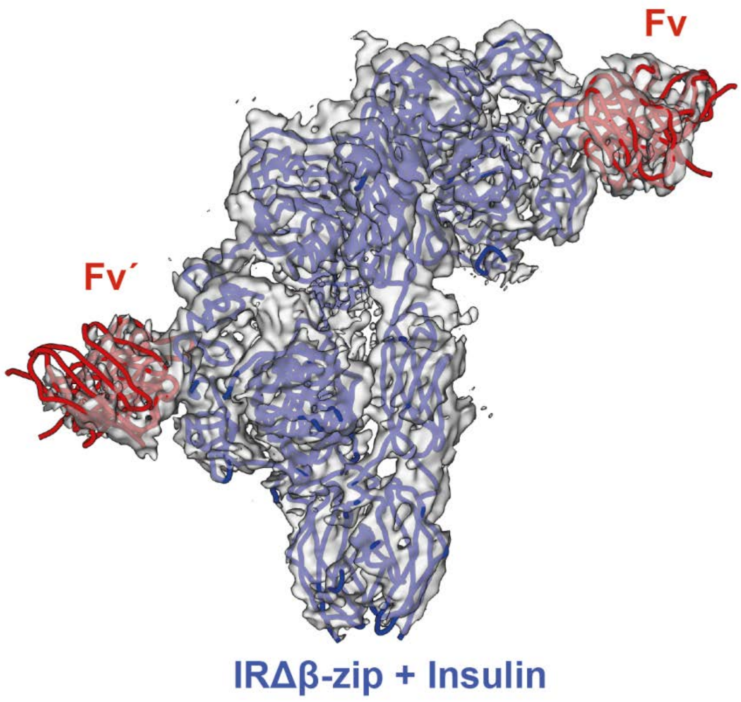

Supplementary Figure 4: Density within the initial map that is associated with the respective Fv 83-7 moieties. The transparent grey map represents the initial 3D reconstruction of IRΔβ-zipInsFv prior to focused refinement of upper and lower volumes of the map. The blue ribbon depicts insulin-bound IRΔβ-zip and the red ribbon the attached Fv 83-7 moieties. The latter are positioned here by overlay of the crystal structure of the complex of the insulin-receptor L1-CR module, Fab 83-7, insulin and exogenous αCT directly onto the CR domains of the cryoEM structure determined here. While the spatial correspondence of the Fv 83-7 modules with the map density is apparent, associated map volumes were relatively poor in quality and the two Fv volumes were thus excluded from the focused refinement.

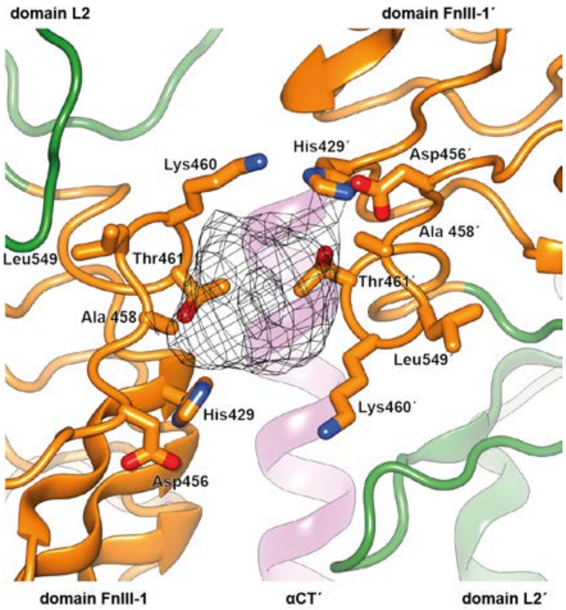

Supplementary Figure 5: Density feature near His429, His 429', Lys460 and Lys460' as seen within the *B*-factor-sharpened upper map. The feature (hollow) is contoured here at 0.071 units, with the contoured volume being a 7 Å radius sphere centred on the approximate geometric of mass of the feature. The peak density within the feature is 0.151 units, with the highest map density being 0.216 units (in the vicinity of the Cα atom of IRΔβ-zip residue Leu62, within domain L1).

1	HLYPGEVCPGMDIRNNLTRLHELENCSVIEGHLQILLMFKTRPEDFRDLS	50
51	FPKLIMITDYLLLFRVYGLESLKDLFPNLTVIRGSRLFFNYALVIFEMVH	100
101	LKELGLYNLMNITRGSVRIEKNNELCYLATIDWSRILDSVEDNHIVLNKD	150
151	DNEECGDICPGTAKGKTNCPATVINGQFVERCWTHSHCQKVCPTICKSHG	200
201	CTAEGLCCHSECLGNCSQPDDPTKCVACRNFYLDGRCVETCPPPYYHFQD	250
251	WRCVNFSFCQDLHHKCKNSRRQGCHQYVIHNNKCIPECPSGYTMNSSNLL	300
301	CTPCLGPCPKVCHLLEGEKTIDSVTSAQELRGCTVINGSLIINIRGGNNL	350
351	AAELEANLGLIEEISGYLKIRRSYALVSLSFFRKLRLIRGETLEIGNYSF	400
401	YALDNQNLRQLWDWSKHNLTTTQGKLFFHYNPKLCLSEIHKMEEVSGTKG	450
451	RQERNDIALKTNGDKASCENELLKFSYIRTSFDKILLRWEPYWPPDFRDL	500
501	LGFMLFYKEAPYQNVTEFDGQDACGSNSWTVVDIDPPLRSNDPKSQNHPG	550
551	WLMRGLKPWTQYAIFVKTLVTFSDERRTYGAKSDIIYVQTDATNPSVPLD	600
601	PISVSNSSSQIILKWKPPSDPNGNITHYLVFWERQAEDSELFELDYCLKG	650
651	LKLPSRTWSPPFESEDSQKHNQSEYEDSAGECCSCPKTDSQILKELEESS	700
701	FRKTFEDYLHNVVFVPRPSRKRRSLGDVGNAGNN................	750
751	...EEHRPFEKVVNKESLVISGLRHFTGYRIELQACNQDTPEERCSVAAY	800
801	VSARTMPEAKADDIVGPVTHEIFENNVVHLMWQEPKEPNGLIVLYEVSYR	850
851	RYGDEELHLCVSRKHFALERGCRLRGLSPGNYSVRIRATSLAGNGSWTEP	900
901	TYFYVTDYLDVPSNIARMKQLEDKVEELLSKNYHLENEVARLKKLVGER	949

Supplementary Table 1: Protein sequence of IRΔβ-zip[a].

[a]The overall residue numbering corresponds to that of the IR-A receptor isoform. The 33-residue C-terminal GCN4 zipper component is underlined, the "Δβ" modification[3] is indicated by double underlining for mutated residues and "." symbols for deleted residues. Population variant residues with respect to the reference sequence, *viz.*, Tyr144His, Ile421Thr and Gln465Lys, are boxed[4].

	"Upper" map[a] (EMDB-0247) (PDB 6HN5)	"Lower" map (EMDB-0246) (PDB 6HN4)
**Data collection and processing**		
Voltage (kV)	300	300
Electron exposure (e–/Å^2)	37	37
Defocus range (μm)	-1.0 to -2.5	-1.0 to -2.5
Pixel size (Å)	1.04	1.04
Symmetry imposed	C1	C1
Final particle images (no.)	213,867	98,481
Map resolution (Å)	3.2	4.2
Map resolution range (Å)	2.5 to 4.5	3.5 to 5.5
**Refinement**		
Map sharpening *B* factor (Å^2)	-107.8	-174.2
Model composition		
Non-hydrogen atoms	7863	5702
Protein residues	955	700
Ligands	11	6
R.m.s. deviations		
Bond lengths (Å)	0.012	0.007
Bond angles (°)	1.2	1.5
Validation		
MolProbity score	1.98	1.85
Clashscore	7.50	6.18
Poor rotamers (%)	0.12	0.00
Ramachandran plot		
Favored (%)	89.22	91.37
Allowed (%)	10.78	6.87
Disallowed (%)	0.00	1.75

Supplementary Table 2: Statistics of cryo-EM data collection, 3D reconstruction and model building.
[a]The "upper" map encompasses domains L1, CR, L2, FnIII-1, L2', FnIII-1', αCT' and insulin, and the "lower" map domains FnIII-2, FnIII-3, L1', CR', FnIII-2' and FnIII-3', with the polypeptide without the ' symbol being that which contributes domain L1 to the insulin binding site.

Supplementary References

1 Kremer, J.R., Mastronarde, D.N. & McIntosh, J.R. Computer visualization of three-dimensional image data using IMOD. *J. Struct. Biol.* **116**, 71-76 (1996).

2 Kucukelbir, A., Sigworth, F.J. & Tagare, H.D. Quantifying the local resolution of cryo-EM density maps. *Nat. Methods* **11**, 63-65 (2014).

3 McKern, N.M. *et al.* Structure of the insulin receptor ectodomain reveals a folded-over conformation. *Nature* **443**, 218-221 (2006).

4 Ebina, Y. *et al.* The human insulin receptor cDNA: the structural basis for hormone-activated transmembrane signalling. *Cell* **40**, 747-758 (1985).
